# Editorial: ****Co-Evolution of Plant Cell Wall Polymers

**DOI:** 10.3389/fpls.2020.598299

**Published:** 2020-09-18

**Authors:** Elisabeth Jamet, Christophe Dunand, Zoë A. Popper

**Affiliations:** ^1^ Laboratoire de Recherche en Sciences Végétales, Université de Toulouse, CNRS, UPS, Auzeville-Tolosane, France; ^2^ Botany and Plant Science, School of Natural Sciences, National University of Ireland Galway, Galway, Ireland; ^3^ Ryan Institute for Environmental, Marine, and Energy Research, School of Natural Sciences, National University of Ireland Galway, Galway, Ireland

**Keywords:** algae, arabinogalactan protein, cell wall, charophyte, gametophyte, grass, land plant, terrestrialization

Possessing a cell wall, composed of different types of polymers including polysaccharides, lignins, and proteins, is one of the defining features of plant and algal cells (Carpita and Gibeaut, 1993; Neutelings, 2011). It has been instrumental in the ability of a single lineage within the streptophyte algae to successfully transition to life on land in the mid-Paleozoic and subsequently to diversify to form the entire terrestrial macroflora (de Vries and Archibald, 2018). Cell walls provide mechanical support to the plant and constitute a physical protective barrier against the environment (Niklas et al., 2017). They also enable cell-to-cell communication (Tavormina et al., 2015; De Lorenzo et al., 2019; Shi et al., 2019) and are modified in a dynamic manner during plant development and in response to biotic and abiotic stresses (Frankova and Fry, 2013; Le Gall et al., 2015; Cosgrove, 2018; Herger et al., 2019). Their structural organization and composition display a large diversity between species and between organs, and even cells, of a single species (Freshour et al., 1996; Sarkar et al., 2009; Popper et al., 2011).

Since the emergence of land plants, important physiological, morphological and environmental changes have occurred corresponding to major cell wall innovations (Graham et al., 2000). Three major innovations can be highlighted: (i) the evolution of multicellularity, and change in body plan to a three-dimensional, rather than planar or filamentous, form, leading to the requirement for cell adhesion and cell-to-cell communication; (ii) the appearance of a composite cuticle made of several layers of polysaccharides, wax, and cutin to protect cells against UV light and desiccation; and (iii) the addition of aromatic compounds eventually polymerized into lignins to waterproof the walls of conducting vessels and reinforce walls, thus allowing the growth in height of vascular plants. The adaptability of cell wall components is, in part, inherently enabled by being carbohydrate-based. For example, Prestegard et al. (2017) were able to construct 1,792 distinct tetrasaccharides from a single sugar in ring form, and there are more than 10 major sugar residues that are commonly found in plant cell wall components, each of which can occur in multiple forms. The evolution and diversity of cell wall polysaccharides and aromatic compounds have necessarily been accompanied by the diversification of cell wall structural proteins and wall-localized remodeling enzymes and of proteins contributing to the biosynthesis of cell wall constituents. Biosynthesis of pectin alone, a highly structurally diverse carbohydrate, is hypothesized to require the activities of at least 67 distinct enzymes (Atmodjo et al., 2013).

Tools for molecular analyses and genomic data are now available and allow more integrative studies to better understand the evolution and co-evolution of cell wall polysaccharides, aromatic compounds, and proteins in the context of multicellularity and terrestrialization. This Research Topic has collected eight original contributions, including two reviews and six original research articles.

Interestingly, and perhaps because of the key role of the cell wall in terrestrialization, three articles focus on the extracellular matrix of Charophytes, the extant group of green algae that are most closely related to living land plants. Fitzek et al. have analyzed and assembled the transcriptome of *Zygnema* *circumcarinatum*. They have compared three glycosyl transferase families (GT2, GT8, and GT43) involved in the biosynthesis of cell wall polysaccharides in eight charophytes including the *Zygnematophyceae*, with those of five embryophytes, and two chlorophytes used as outgroups. Regarding the GT2 family, orthologs to the land plant cellulose synthases (CesA) were only found in *Zygnematophyceae* and not in the seven other charophytes nor in the two chlorophytes, whereas the cellulose synthases-like (Csl) genes detected in all the charophytes form a distinct cluster. Interestingly, in response to osmotic stress, the *Z. circumcarinatum* genes encoding ZcCesA (cellulose synthase), ZcCslC (possibly mannan synthase), and ZcCslA-like (possibly xyloglucan synthase) were induced as is found in land plants. Herburger et al. have focused their study on the pectic cell wall polysaccharides, and particularly on homogalacturonans (HG). They show that the accumulation of HG in *Z. circumcarinatum* filaments increases their resistance to dessication. This feature could have played a role during colonization of the land by the *Zygnematophyceae*. Palacio-López et al. highlight the importance of arabinogalactan proteins (AGPs) in cell-to-cell and cell-to-surface adhesion in four Charophytes*, Z. circumcarinatum*, *Penium margaritaceum*, *Chlorokybus atmophyticus*, and *Coleochaete orbicularis*. They hypothesize that effective adhesion mechanisms would likely have supported land colonization because they are an asset in highly changeable wetlands and that a stable position on a substratum increases light absorption and favors water movement, helping combat dessication.

Dehors et al. provide a review article on the evolution of cell wall polymers in the tip-growing gametophytes of land plants. A massive deposition of cell wall material is required to support rapid elongation of tip-growing structures like pollen tubes as well as a tight control of the remodeling of the cell wall to ensure its rigidity. The authors review the diversity of the cell wall polymers, which contribute to the growth expansion of gametophytes from basal to later diverging land plants.

Two articles are devoted to grass cell walls which show particularities with the presence of (1,3; 1,4)-β-glucans and arabinoxylans as hemicelluloses and reduced amounts of pectins, mannans, and xyloglucans when compared with other vascular plants. Bulone et al. question the origin of (1,3; 1,4)-β-glucan synthases from synthases originally only synthesizing (1,4)-β-linkages. In parallel, (1,3; 1,4)-β-glucan endohydrolases evolved presumably from (1,3)-β-glucan endohydrolases. Penning et al. focus on the genes encoding proteins involved in the biosynthesis of the cell wall polysaccharides. They show that, unexpectedly, genes associated with the biosynthesis of pectins and xyloglucans are transcribed in *Zea mays*, considered as a model for grasses. After combining these transcriptomics results to a previous proteomics study, they propose that the specific composition of grass cell walls results from late sorting events occurring in the Golgi and at a post-Golgi step.

Finally, two articles are devoted to secondary walls. Chernova et al. explore the composition of the cell walls of a fern, *Psilotum nudum*, cortical fibers to understand the emergence and evolution of secondary walls. The composition of the secondary walls of the fibers is enriched in mannans as that of primary walls, but different from that of wall of tracheids. In parallel, they also show that the composition of secondary walls varies in early land plants. Blaschek et al. look at the incorporation of coniferaldehyde in lignin in herbaceous (*Arabidopsis thaliana*) and in woody (hybrid poplar) plants. They show that, in both plants, this incorporation is controlled by autonomous biosynthesis pathways for each cell type, cell-to-cell cooperation, and cell wall layer–specific accumulation capacity.

## Author Contributions

EJ, ZP, and CD wrote the editorial.

## Conflict of Interest

The authors declare that the research was conducted in the absence of any commercial or financial relationships that could be construed as a potential conflict of interest.
